# Immune checkpoint inhibitor-induced colitis with endoscopic evaluation in Chinese cancer patients: a single-centre retrospective study

**DOI:** 10.3389/fonc.2023.1285478

**Published:** 2023-11-28

**Authors:** Furong Kou, Jian Li, Yanshuo Cao, Zhi Peng, Ting Xu, Lin Shen, Jifang Gong, Xicheng Wang

**Affiliations:** ^1^ Department of Gastrointestinal Oncology, Key Laboratory of Carcinogenesis and Translational Research (Ministry of Education/Beijing), Peking University Cancer Hospital & Institute, Beijing, China; ^2^ Key Laboratory of Carcinogenesis and Translational Research (Ministry of Education), Department of Comprehensive Clinical Trial Ward, Peking University Cancer Hospital & Institute, Beijing, China; ^3^ Key Laboratory of Carcinogenesis and Translational Research (Ministry of Education), Early Drug Development Center, Peking University Cancer Hospital & Institute, Beijing, China

**Keywords:** immune checkpoint inhibitor, colitis, endoscopy, steroid, infliximab, rechallenge

## Abstract

**Background:**

We investigated the clinical and endoscopic features, management strategies, and outcomes of Chinese cancer patients with immune checkpoint inhibitor (ICI)-induced colitis.

**Method:**

This single-centre retrospective study included patients who developed ICI-induced colitis and underwent endoscopic evaluation from June 1, 2019 to October 1, 2023. We analysed clinical features, ICI-induced colitis-related information, management strategies, and outcomes.

**Results:**

A total of 25 patients were included; most were male (88%) with a median age of 59 years. Eleven (44%) patients had grade 2 colitis, and 14 (56%) had grade 3 colitis. The median time from ICI initiation to colitis onset was 105 days. The median duration from symptom onset to endoscopic evaluation was 11 days. Regarding endoscopic evaluation, colitis involved the entire colon in 13 (52%) patients, and 15 (60%) had ulcers. Twenty-three (92%) patients received steroids, and 3 (12%) added infliximab (IFX). Most patients (n=19, 76%) achieved remission with complete tapering of the steroid taken for the first colitis episode. Among the 6 (24%) patients who did not taper initial, 5 patients increased their steroid dosage with 2 added IFX, leading to symptom remission and successful steroid tapering, while one patient experienced continuous non-remission despite increasing the steroid and receiving two infusions of IFX. Of the 8 (32%) ICI rechallenge patients, 4 achieved long-lasting benefit without colitis recurrence. The other 4 experienced recurrent colitis after ICI rechallenge and permanently discontinued ICIs. The median duration from ICI rechallenge to colitis recurrence was shorter than the time to colitis onset. One patient developed steroid-refractory colitis and recovered with one infusion of IFX.

**Conclusion:**

Endoscopy has value in the evaluation and optimal management of ICI-induced colitis in Chinese cancer patients. IFX is necessary for treating colitis, especially in steroid-refractory/resistant patients. ICI rechallenge can achieve benefit, but permanently discontinuing ICIs is needed if colitis recurs. Future large-scale prospective studies are required for more accurate assessments and validation.

## Introduction

Immune checkpoint inhibitors (ICIs), such as programmed death-1/ligand 1 (PD-1/L1) inhibitors and cytotoxic T-lymphocyte-associated protein-4 (CTLA-4) inhibitors, are promising drugs that can enhance the immune system for cancer treatment (1). ICIs have shown significant potential in improving overall survival (OS) in various cancers, such as melanoma, non-small-cell lung carcinoma, and renal cell carcinoma (2). ICIs have revolutionized the management of advanced cancer therapeutics.

Despite the significant clinical benefits of ICIs, immune-related adverse events (irAEs) occur in association with ICI treatment due to activation of T cells. Commonly observed toxicities can affect the skin, endocrine organs, gastrointestinal tract, liver, and lungs (3, 4). ICI-induced colitis is one of the most common and serious irAEs and is also the main reason for immunotherapy discontinuation (5). A meta-analysis reported that the incidence of grade 3~4 colitis was 9.1% with CTLA-4 monotherapy, 1.3% with PD-1/L1 therapy, and 13.6% with combination therapy (6).

Considering its potentially fatal outcomes, early detection and appropriate management of ICI-induced colitis are needed. Endoscopic and histological findings could provide valuable insights into the optimal management of ICI-induced colitis (7, 8). Because irAEs are autoimmune entities, they should be reversable by immunosuppression. Current guidelines recommend steroids as first-line treatment, followed by selective immunosuppressive therapy (SIT) for steroid-refractory/resistant or severe cases. Infliximab (IFX) and vedolizumab (VDZ) are the two commonly used SITs for ICI-induced colitis (9–11). IFX, as a chimeric antitumour necrosis factor-alpha (TNFα) monoclonal antibody, can broadly dampen TNFα’s role in the immune response. Unlike IFX, VDZ is a gut-selective humanized anti-α4β7 monoclonal antibody, thus preventing lymphocyte infiltration into the gut.

Given that immunotherapy in China started later than in Western countries, we could find no paper that has described the clinical course of ICI-induced colitis from the perspective of cancer patients in China, let alone the endoscopic evaluation of colitis. The Chinese population might differ in susceptibility compared with Western ones. The aim of our study, in which we describe our experience as a major Chinese cancer centre, was to investigate the clinical and endoscopic features, management strategies, and outcomes among Chinese cancer patients who developed colitis induced by various ICIs.

## Materials and methods

### Study design and population

This was a retrospective single-centre study. We investigated adult cancer patients who were treated with ICIs at Peking University Cancer Hospital between June 1, 2019 and October 1, 2023.

Patients were eligible for inclusion if they had received at least 1 cycle of any ICI, including PD-1/L1 inhibitors and CTLA-4 inhibitors, as a single agent or in combined therapy within a clinical trial or otherwise. Other inclusion criteria were patients who developed ICI-induced colitis and underwent endoscopic evaluation.

We excluded patients who had autoimmune disease including inflammatory bowel disease (IBD) or received transplantation before ICIs treatment. Besides, various methods including past medical history, blood/stool test (such as complete blood count, erythrocyte sedimentation rate, C-reactive protein, virus detection, stool cultures and ova and parasites testing), endoscopic examination and response to treatment were used to determine the origin of colitis. We excluded patients with colitis due to other etiologies, including infectious colitis, neutropenic colitis, inflammatory bowel disease, graft-versus-host disease, etc.

The study was approved by the medical ethics committee of Peking University Cancer Hospital and was conducted in accordance with the Declaration of Helsinki.

### Information collection

Electronic medical records were retrospectively investigated to collect data, including patient demographics and cancer characteristics, ICI-induced colitis-related information, treatment and outcomes.

#### Patients and cancer characteristics

Pertinent variables were collected regarding age, sex, Eastern Cooperative Oncology Group (ECOG) score, cancer type and stage (based on American Joint Committee on Cancer Staging System, 7th edition), type of ICIs and lines of immunotherapy.

#### ICI-induced colitis-related information

The severity of colitis at the time of diagnosis, graded as 1~5, was assessed using the Common Terminology Criteria for Adverse Events version 5.0 (CTC-AE V5.0), which was characterized as below: grade 1: asymptomatic, clinical or diagnostic observations only, intervention not indicated; grade 2: abdominal pain, mucus or blood in stool; grade 3: severe abdominal pain, peritoneal signs; grade 4: life-threatening consequences, urgent intervention indicated; grade 5: death. The duration of symptoms was measured from the time of symptom onset to resolution. We also calculated the time from ICI initiation to colitis onset.

Data relating to endoscopy included gross presentation and colitis distribution. The distribution of colitis was divided into terminal ileum involvement with or without colon involvement, left colon involvement only, right colon involvement only, and entire colon involvement. Gross presentation on endoscopy was characterized as the presence of mucosal ulcers or no. The time from symptom onset to the first endoscopic evaluation was also recorded. Histopathological evaluations were classified as active histological inflammation and chronic inflammation if biopsies were obtained.

#### Treatment and outcome assessment

Agents used for ICI-induced colitis were steroids and SITs (IFX or VDZ). Infection, as a common complication of immunosuppressive treatment, was recorded. The duration of steroid use was defined as the initiation of steroids used for colitis to the time of cessation. We used 30 days as the cut-off to indicate short duration (≤30 days) and long duration (>30 days) of steroid use. Clinical outcomes included clinical/endoscopic remission with complete steroid tapering and failed steroid tapering. Steroid tapering was defined as failed if the symptoms recurred after reducing the dose of steroids from the initial dose. The information gathered on the immunotherapy rechallenge population included rechallenge regimens, recurrence of colitis, and treatment of colitis recurrence.

### Statistical analysis

Statistical analyses were carried out using SPSS version 24.0 software (SPSS, Inc., Chicago, IL). Continuous variables are summarized using the median and range and compared using the Wilcoxon rank-sum test. The distribution of categorical variables is presented as frequencies and percentages and compared by Fisher’s exact and chi-squared test. Statistical significance was defined as a two-sided P value < 0.05.

## Results

### Patient characteristics

A total of 25 patients who developed ICI-induced colitis and underwent endoscopic evaluation were included in our analysis. The mean age was 59 years (range, 45~79 years). The predominant sex was male (n=22, 88%) and predominant ECOG score 0~1 (n=23, 92%). The most common cancer types were melanoma (n=5, 20%) and gastric adenocarcinoma (n=5, 20%). Most patients had stage IV malignancy (n=22, 88%).

Concerning ICI therapy, 22 (88%) patients received PD-1 inhibitor-based therapy: monotherapy (n=3, 12%) or combination therapy (n=19, 76%). The combination agents consisted of chemotherapy drugs, targeted drugs, CTLA-4 inhibitors and T-cell Ig and ITIM domain (TIGIT) inhibitors. The other 3 (12%) patients received PD-L1 based dual inhibitor, including PD-L1/transforming growth factor-β receptor type II (TGF-β RII) dual inhibitor (n=2) and PD-L1/vascular endothelial growth factor (VEGF) inhibitor (n=1). Most patients received ICI treatment as first-line therapy (n=14, 56%), followed by second-line therapy (n=8, 32%). Eight (32%) patients received ICIs in clinical trials.

Patient characteristics in the entire population are shown in **Table 1**.

**Table 1:** Patient characteristic in the entire population (n=25).

Characteristic	n (%)
Age, median (range), years	59 (45~79)
Gender	
Male	22 (88)
Female	3 (12)
ECOG score	
0~1	23 (92)
2	2 (8)
Cancer type	
Melanoma	5 (20)
Gastric adenocacinoma	5 (20)
Oesophageal squamous cell carcinoma	4 (16)
Lung cancer	3 (12)
Head and neck squamous cell carcinoma	2 (8)
Sigmoid colon adenocarcinoma	1 (4)
Pancreatic adenocarcinoma	1 (4)
Cholangiocarcinoma	1 (4)
Ureteral urothelial carcinoma	1 (4)
Thymic squamous cell carcinoma	1 (4)
Primary unknown neuroendocrine carcinoma	1 (4)
Cancer stage	
Stage II	1 (4)
Stage III	2 (8)
Stage IV	22 (88)
Types of ICI	
PD-1 inhibitor	3 (12)
PD-1 inhibitor + Chemotherapy	6 (24)
PD-1 inhibitor + Targeted therapy^a^ +/- Chemotherapy	8 (32)
PD-1 inhibitor + CTLA-4 inhibitor	4 (16)
PD-1 inhibitor + TIGIT inhibitor	1 (4)
PD-L1/TGF-β RII inhibitor+/-Chemotherapy^b^	2 (8)
PD-L1/VEGF inhibitor	1 (4)
Lines of immunotherapy	
Adjuvant/Neoadjuvant therapy	2 (8)
First-line therapy	14 (56)
Second-line therapy	8 (32)
Third-line therapy	1 (4)
Receiving immunotherapy in clinical trials	8 (32)

ECOG, Eastern Cooperative Oncology Group; ICI, immune checkpoint inhibitor; CTLA-4, cytotoxic T-lymphocyte antigen-4; PD-1/L-1, programmed cell death receptor-1 and ligand 1; TIGIT, T cell Ig and ITIM domain; TGF-β RII, transforming growth factor-β receptor type II; VEGF, vascular endothelial growth factor.

a Targeted therapy including: anlotinib, afatinib, axitinib, apatinib, bevacizumab, trastuzumab, RC48-ADC.

b PD-L1/TGF-β RII monotherapy or combined with chemotherapy: each 1 case.

### ICI-induced colitis features

Diarrhoea was the most common symptom (n=22, 88%) patients. The diarrhoea was mostly described as watery, occurring 4~20 times daily, while 10 (40%) patients had mucous stool/bloody stool. The median duration of symptoms was 31 days (range, 4~122 days). According to colitis grade, 11 (44%) patients had grade 2 and 14 (56%) patients had grade 3. Seven (28%) patients had other organ-specific irAEs: hypothyroidism/hyperthyroidism (n=4), adrenocortical insufficiency (n=2), hepatitis (n=1), myositis (n=1), and pancreatitis (n=1). The median time from ICI initiation to colitis onset was 105 days (range, 4~582 days). The median number of immunotherapy cycles was 6 (range, 1~21).

**Figure 1:**
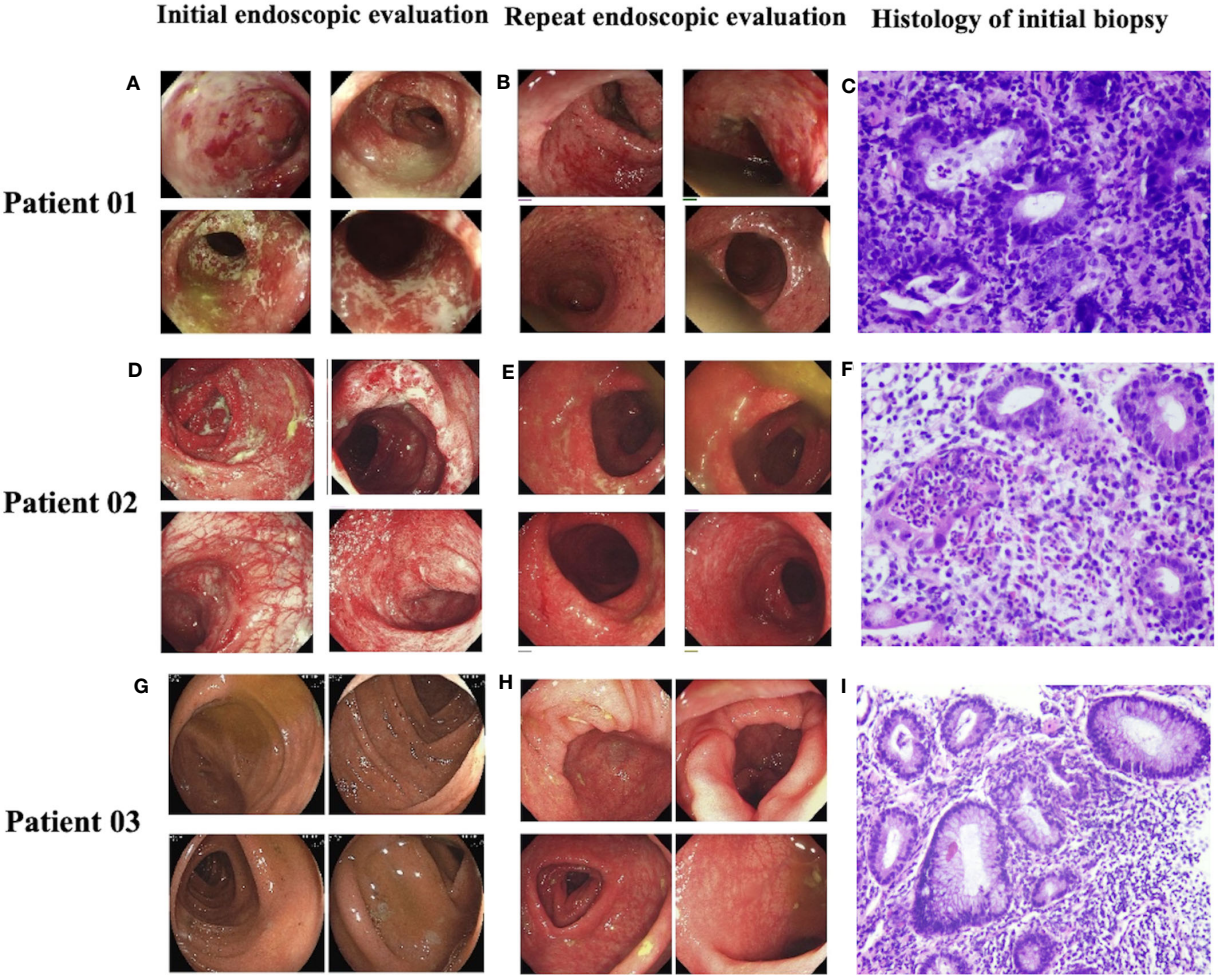
Endoscopic and histologic images in 3 patients. Initial endoscopic evaluation showed multiple superficial ulcers and a edematous and erosive mucosa **(A, D, G)**. Repeat endoscopic evaluation after treatment showed edema and erosion of mucosa became better **(B, E, H)**. Hematoxylin-eosin stained (HE) sections of the initial biopsy showed inflammation with neutrophil infiltration and cryptitis **(C, F, I)**.

The median duration from symptom onset to endoscopic evaluation was 11 days (range, 0~90 days). Regarding the distribution of colitis as evaluated by endoscopy, the colitis involved the entire colon in 13 (52%) patients, with 4 (16%) patients having terminal ileum involvement. In 11 (44%) patients, colitis was limited to the left colon, whereas in one (4%) patient was it limited to the right colon. Regarding gross presentation on endoscopy, 15 (60%) patients had ulcers, and 10 (40%) had no ulcers. Fifteen (44%) patients underwent biopsy, while most patients (n=11) had features of active inflammation on histology (**Table 2**). Patients` endoscopic and histologic images were shown in **Figure 1**.

**Table 2:** ICI-induced colitis features in all patients (n=25).

Characteristic	n (%)
Symptoms	
Diarrhoea	22 (88)
Mucous stool/bloody stool	10 (40)
Abdominal pain	7 (28)
Duration of symptom, median (range), days	31 (4~122)
Grade of colitis	
Grade 2	11 (44)
Grade 3	14 (56)
Combined with other organ-specific irAEs	
Hypothyroidism/hyperthyroidism	4 (16)
Adrenocortical insufficiency	2 (8)
Hepatitis	1 (4)
Myositis	1 (4)
Pancreatitis	1 (4)
Duration from ICI initiation to colitis onset, median (range), days	105 (4~582)
Cycles of immunotherapy, median (range), cycles	6 (1~21)
1~3	10 (40)
4~9	9 (36)
≥10	6 (24)
Duration from symptom onset to endoscopic evaluation, median (range), days	11 (0~90)
Distribution of colitis evaluated by endoscopy	
Terminal ileum involved	4 (16)
Right colon only	1 (4)
Left colon only	11 (44)
Entire colon	13 (52)
Gross presentation on endoscopy	
Ulcers	15 (60)
No ulcers	10 (40)
Histological features of biopsies	
Active histological inflammation	11 (44)
Chronic inflammation	4 (16)
No biopsy	10 (40)
Repeat endoscopic evaluation	9 (36)

ICI, immune checkpoint inhibitor; irAE, immune-related adverse events.

### Treatment and outcomes of ICI-induced colitis

As for the treatment of ICI-induced colitis, 20 (80%) received steroid therapy without SIT, and 3 (12%) added IFX to the steroid. All patients were treated symptomatically, such as with montmorillonite powder, loperamide, mesalazine and probiotics. Intravenous steroids were needed in 18 (72%) patients. The duration of steroid use was short in 7 (28%) patients and long in 18 (72%). The three patients who received IFX received one or two infusions with 300 mg per infusion (one infusion, n=1; two infusions, n=2). Their time from steroid initiation to the introduction of IFX was 5 days, 26 days and 35 days, respectively. We found immunosuppressant (steroid and IFX)-associated infections in 2 (8%) patients.

In our cohort, 19 (76%) patients achieved clinical remission with complete tapering of steroids for the first colitis episode, while 9 (36%) patients underwent repeat endoscopic evaluation with evidence of remission. Another 6 (24%) patients failed steroid tapering during initial treatment. Among them, 5 patients increased the steroid dosage, 2 of them adding IFX in the following treatment regimen, leading to symptom remission and successful steroid tapering. However, one patient experienced continuous non-remission despite increasing the steroid and receiving two infusions of IFX.

The treatment and outcomes of ICI-induced colitis are described in detail in **Table 3**.

**Table 3:** Treatment and outcomes of ICI-induced colitis (n=25).

Characteristic	n (%)
Treatment of ICI-induced colitis	
No steroids	2 (8)
Steroids	20 (80)
Steroids + Infliximab	3 (12)
Intravenous steroids	18 (72)
Overall duration of steroid use, median (range), days	39 (0~118)
Short duration (≤ 30days)	7 (28)
Long duration (> 30days)	18 (72)
Immunosuppressant-associated infection	2 (8)
Outcomes	
Clinical/Endoscopic remission with complete steroid tapering	19 (76)
Failed steroid tapering initially	6 (24)
Response after increasing steroid dosage or adding infliximab	5 (20)
Continuous non-remission	1 (4)

ICI, immune checkpoint inhibitor.

### ICI rechallenge

ICI therapy was rechallenged in 8 (32%) patients after initial colitis resolution due to the good response to ICIs, while the remaining 17 (68%) patients discontinued ICI treatment. No significant differences were observed in patient characteristics, ICI-induced colitis features or management strategies between the two groups (**Table 4**). Of the 8 rechallenge patients, 5 (63%) received their original regimens again, and 3 (37%) patients adjusted them, including switching dual ICIs to monotherapy (sintilimab+ipilimumabsintilimab, n=1), changing the combination agent (pembrolizumab+ chemotherapypembrolizumab+anlotinib, n=1), and replacing the type of ICI (camrelizumab+afatinibtislelizumab+afatinib, n=1). Overall, 3 (37%) patients received PD-1 inhibitors, whereas the other 5 (63%) patients used combination therapy.

**Table 4:** Characteristics in population with ICI-rechallenge or not (n=25).

Characteristic	Rechallenge population, n=8	Non-rechallenge population, n=17	*P* value
Age, median (range), years	56 (46~73)	60 (45~79)	0.066
Gender, n (%)			0.968
Male	7 (87)	15 (88)	
Female	1 (13)	2 (12)	
ECOG score, n (%)			0.312
0~1	8 (100)	15 (88)	
2	0 (0)	2 (12)	
CTLA-4 inhibitor containing, n (%)			0.400
Yes	2 (25)	2 (12)	
No	6 (75)	15 (88)	
Grade of colitis, n (%)			0.201
Grade 2	5 (63)	6 (35)	
Grade 3	3 (37)	11(65)	
Duration of symptom, median (range), days	29 (18~34)	32 (4~122)	0.238
Duration from ICI initiation to colitis onset, median (range), days	81 (20~395)	112 (4~582)	0.932
Duration from symptom onset to endoscopic evaluation, median (range), days	13 (5~90)	9 (0~53)	0.588
Distribution of colitis evaluated by endoscopy, n (%)			0.064
Left/Right colon only	6 (75)	6 (35)	
Entire colon	2 (25)	11 (65)	
Gross presentation on endoscopy, n (%)			0.484
Ulcers	4 (50)	6 (35)	
No ulcers	4 (50)	11 (65)	
Overall duration of steroid use, median (range), days	37 (3~51)	57 (0~118)	0.288
Introduction of Infliximab			0.205
Yes	0 (0)	3 (18)	
No	8 (100)	14 (82)	

ECOG, Eastern Cooperative Oncology Group; ICI, immune checkpoint inhibitor; CTLA-4, cytotoxic T-lymphocyte antigen-4.

Among the 8 rechallenge patients, 4 (50%) achieved long-lasting benefit without recurrence of colitis (treatment time ranging from 2 to 36 months at the time of data cutoff: October 1, 2023). The other 4 (50%) patients experienced recurrent colitis after rechallenge (grade of colitis: grade 2, n=2; grade 3, n=2). The symptoms lasted a median of 41 days (range, 21~69 days). The median time from rechallenge to colitis recurrence was 11 days (range, 2~50 days), which was significantly earlier than the initial colitis onset (*P*=0.008). These patients received only 1~4 cycles of ICI treatment before colitis recurrence. One of the four patients evaluated by endoscopy presented with entire colon inflammation.

Regarding the treatment of colitis in the four patients with colitis recurrence, 1 patient only received symptomatic treatment, and 2 (25%) patients received steroids with successful tapering. One patient developed steroid-refractory colitis and recovered with one infusion of IFX (introduction time of IFX: 46 days). These four patients permanently discontinued ICI treatment due to colitis recurrence.

Patient characteristics and recurrence features in the ICI rechallenge population are shown in **Table 5**.

**Table 5:** Patient characteristics and recurrence of colitis features in ICI re-challenge population (n=8).

Characteristic	n (%)
Immunotherapy re-challenge regimens	
Original regimens	5 (63)
Adjustment regimens	3 (37)
Types of ICI	
PD-1 inhibitor	3 (37)
PD-1 inhibitor + Targeted therapy	2 (25)
PD-1 inhibitor + CTLA-4 inhibitor	1 (13)
PD-L1/TGF-β RII inhibitors+/-Chemotherapy	2 (25)
Recurrence of colitis after re-challenge	4 (50)
Grade of colitis	
Grade 2	2 (25)
Grade 3	2 (25)
Duration of symptoms, median (range), days	41 (21~69)
Duration from ICI re-challenge to colitis recurrence, median (range), days	11 (2~50)
Cycles of immunotherapy before colitis recurrence, median (range)	1 (1~4)
Endoscopic evaluation	1 (13)
Treatment of colitis	
Symptomatic treatment only	1 (13)
Steroids	2 (25)
Steroids + Infliximab	1 (13)

ICI, immune checkpoint inhibitor; CTLA-4, cytotoxic T-lymphocyte antigen-4; PD-1/L-1, programmed cell death receptor-1 and ligand 1; TGF-β RII, transforming growth factor-β receptor type II.

## Discussion

This was the first study to analyse the characteristics of ICI-induced colitis with endoscopic evaluation in Chinese cancer patients. Due to the later advent of immunotherapy in China, approximately one-third of patients in our study were in clinical trials. Most clinical trials were early-phase studies (phase 1, n=4; phase 1b/2, n=1; phase 2, n=3) related to various new experimental drugs, such as a PD-L1/TGF-β RII inhibitor (SHR-1701), a PD-L1/VEGF inhibitor (PM8002), a TIGIT inhibitor (BGB-A1217) and new CTLA-4 inhibitors (HMB-4003, IBI-310).

ICI-induced colitis has a variable presentation, watery diarrhoea being the most common symptom (5). Consistent with this, 88% of patients in our study had diarrhoea, mostly described as watery. Our study showed that the median duration from ICI initiation to colitis onset was 105 days, and the median number of ICI treatments was 6 cycles. In addition, colitis occurred much earlier in CTLA-4 inhibitor-containing regimens than in non-CTLA-4 inhibitor-containing regimens (median duration: 28 days vs. 157 days, *P*=0.019; median immunotherapy cycles: 2 cycles vs. 8 cycles, *P*=0.015). Previous studies have reported the median time duration of developing ICI-induced colitis was 80~110 days with shorter duration for CTLA-4 inhibitors than PD-1/L1 inhibitors in Western population, which was in accordance with our study (8, 12, 13).

Prior studies have suggested the importance of predicting outcomes by assessing disease severity endoscopically and risk-stratifying patients to guide future therapy. Ulcers and entire colon colitis are high-risk features, which indicate more severe colitis and more often require the addition of IFX (8, 11). Early performance of endoscopy (≤7 days) is recommended due to the potential benefit (7). However, few patients undergo endoscopic evaluation when ICI-induced colitis is suspected in China due to low physician awareness and/or patient refusal. Even so, we collected 25 patients who underwent endoscopic evaluation in our centre and found ulcers and entire colon involvement in 60% and 52% of patients, respectively. Similarly, the presentation of ulcers had been reported in 28%~52% of patients, while extensive colitis involvement in 23%~68% of patients in Western population (8, 12, 13). In addition, our study found that the median duration from symptom onset to endoscopic evaluation was 11 days, longer than the recommendation of 7 days. The main reason might be the relatively low awareness of the benefit of endoscopy. Many patients were not evaluated by endoscopy early after colitis was suspected. Once the symptoms worsened in the later course, endoscopy was performed.

In our study, a majority of (80%) patients were treated with steroids according to recent guideline recommendations, intravenous and long-duration (> 30 days) steroid use each accounted for 72% of our patients (14, 15). In total, most (76%) patients achieved clinical/endoscopic remission with complete steroid tapering. With these data and prior data, steroids are the most common and useful agents for colitis.

Our study found that 6 (24%) patients failed steroid tapering, indicating they were steroid-refractory/resistant. One study showed that the most common steroid-refractory/resistant irAEs were diarrhoea/colitis (53%) (16). Of the six patients who failed tapering, 3 added IFX to steroids, and 2 patients achieved symptom remission and successful steroid tapering, which indicated the benefit of adding IFX. In addition, previous studies have reported that early introduction of IFX (≤10 days) was correlated with favourable clinical outcomes with faster symptom remission (17). Therefore, IFX was recommended for early diagnosis of ICI-induced colitis rather than waiting until failure of steroid therapy. Unfortunately, IFX was limited to cases that were refractory/resistant to steroids in the later course of colitis in the present clinical practice. Hence, the introduction time of IFX, as well as the combined modality of IFX with ICIs, needs to be explored in prospective clinical trials. VDZ is another SIT that can be used to treat ICI-induced colitis. Due to drug availability, VDZ is not extensively used in China, and no patients used VDZ in our study. The safety and efficacy of VDZ should be further investigated in Chinese cancer patients.

Many patients who develop ICI-induced colitis, particularly under combination therapy, might tolerate anti-PD-1 rechallenge well. In our study, 8 (32%) patients received ICI rechallenge, 3 of them receiving adjusted regimens and only 1 receiving PD-1 inhibitor plus CTLA-4 inhibitor. Despite no significant differences in the demographic characteristics, initial colitis features, or steroid/IFX usage between the rechallenge and non-rechallenge groups, our results indicated that the ICI rechallenge group had less grade 3 colitis, a shorter duration of symptoms, less entire colon involvement and ulcers, a shorter overall duration of steroid use and no introduction of IFX. Half of the rechallenge patients achieved long-lasting benefit without recurrence of colitis. Our findings are encouraging and add to the current evidence about ICI resumption. Notably, recurrence of colitis occurred significantly earlier after (re)starting ICI than the initial colitis onset, with a median duration from ICI rechallenge to colitis recurrence of 11 days vs. 105 days (*P*=0.008). One ICI rechallenge patient with steroid-refractory colitis added IFX and achieved remission, which indicated that IFX is still useful in the recurrence of colitis. Selection of a rechallenge population with better efficacy and a lower risk of colitis recurrence might be worthy of exploration in the future.

One retrospective large-scale study included 167 patients with ICI resumption after onset of ICI-induced colitis to assess the rate and risk factors for colitis recurrence (18). The results showed that colitis recurred in 34% of patients; 82% of these patients required immunosuppressive therapy for recurrent colitis, and all required permanent discontinuation of ICI therapy. The risk of colitis recurrence was increased if patients had a longer duration of symptoms and required immune-suppression for initial colitis, whereas resumption of anti–PD-1/L1 led to lower risk than resumption of anti–CTLA-4. Our study did not explore the risk factors for colitis recurrence due to the small sample size (colitis recurrence occurred in only 4 patients).

Hence, Chinese population had some differences compared with Western population. Our preliminary data showed the consistency of duration from ICI initiation to colitis onset and endoscopic features of ulcers or distribution between Chinese population and Western population. However, endoscopic evaluation was relatively less and performed later in Chinese population than that in Western population. Regarding treatment, the most common and useful drugs for colitis are steroids in both two population. But IFX was not introduced in the early course of colitis and other SITs (such as VDZ) are not yet widely used in Chinese population compared with Western population.

Limitations of this study should be noted. First, this was a retrospective study with a relatively small number of patients in a single centre, which might bring intrinsic bias with insufficient statistical power. Second, the use of IFX was limited to steroid-refractory/resistant cases. Other new treatment strategies, such as VDZ, tofacitinib (a Janus kinase inhibitor), ustekinumab (an interleukin-12/23 inhibitor), and faecal microbiota transplantation, were not applied in our study (19–21). Third, our study included patients with various solid tumours, which made it difficult to elucidate any relationship between colitis and tumour response or survival. Therefore, multicentre studies involving more patients are necessary to evaluate the relationship between colitis and OS, assess the safety and efficacy of various treatments, and explore predictive biomarkers for colitis (such as gut microbiota, serum cytokines, and gene expression profiling) in the future.

## Conclusions

This study is by far the largest single-centre study focusing on ICI-induced colitis in Chinese cancer patients with endoscopic evaluation. Our study has shown that endoscopy has value in the evaluation and optimal management of colitis. In addition, the introduction of IFX is necessary for ICI-induced colitis, especially in steroid-refractory/resistant patients. ICI rechallenge after initial colitis can achieve benefit in selective patients; however, permanently discontinued ICIs are needed if colitis recurs. Future prospective studies involving more patients are required for more accurate assessments and validation.

## Data availability statement

The raw data supporting the conclusions of this article will be made available by the authors, without undue reservation.

## Ethics statement

The studies involving humans were approved by medical ethics committee of Peking University Cancer Hospital. The studies were conducted in accordance with the local legislation and institutional requirements. The participants provided their written informed consent to participate in this study.

## Author contributions

FK: Data curation, Methodology, Project administration, Software, Writing – original draft, Writing – review & editing. JL: Investigation, Supervision, Writing – review & editing. YC: Methodology, Project administration, Writing – review & editing. ZP: Software, Supervision, Writing – review & editing. TX: Formal Analysis, Project administration, Writing – review & editing. LS: Supervision, Writing – review & editing. JG: Methodology, Writing – review & editing. XW: Conceptualization, Supervision, Writing – review & editing.
